# Effect of stocking density and alpha-lipoic acid on the growth performance, physiological and oxidative stress and immune response of broilers

**DOI:** 10.5713/ajas.18.0939

**Published:** 2019-04-15

**Authors:** Wenjia Li, Fengxian Wei, Bin Xu, Quanyou Sun, Wen Deng, Huihui Ma, Jie Bai, Shaoyu Li

**Affiliations:** 1 Institute of Animal Husbandry and Veterinary Science, Henan Academy of Agricultural Sciences, Zhengzhou, Henan 450003, China

**Keywords:** Alpha-lipoic Acid, Antioxidative Ability, Broiler, Immune Response, Stocking Density

## Abstract

**Objective:**

The study was conducted to evaluate the effect of stocking density and alpha-lipoic acid (ALA) on the growth performance, feed utilization, carcass traits, antioxidative ability and immune response of broilers.

**Methods:**

A total of 1,530 22-day-old male broilers (*Arbor Acres*) with comparable body weights (731.92±5.26) were placed into 18 cages (2.46×2.02 m) in groups of 75 birds (15 birds/m^2^, 37.5 kg/m^2^; low stocking density [LD]), 90 birds (18 birds/m^2^, 45.0 kg/m^2^; high stocking density [HD]) and 90 birds with 300 mg/kg ALA added to the basal diet (18 birds/m^2^, 45.0 kg/m^2^; HD+ALA, high stocking density+α-lipoic acid); each treatment was represented by 6 replicates. The experimental period was 3 weeks.

**Results:**

The results showed that the high stocking density regimen resulted in a decreased growth, feed conversion ratio, carcass weight, thigh yield and bursa weight relative to body weight (p<0.05) on d 42. The abdominal fat yield in the HD+ALA group was lower (p = 0.031) than that of the LD group at 42 d. The superoxide dismutase and glutathione peroxidase activities in serum were increased, and malondialdehyde content decreased after adding ALA product (p<0.05) on d 42. Additionally, the serum concentrations of immunoglobulin A (IgA) and IgG were decreased (p<0.05) and the level of diamine oxidase was higher (p< 0.01) in the HD group on d 42.

**Conclusion:**

The high stocking density significantly decreased broiler growth performance, feed utilization and carcass traits, increased physiological and oxidative stress and induced intestinal mucosal injury. The supplementation of ALA product in broiler diet at 300 mg/kg may reduce the adverse effects of high stocking density-mediated stress by maintaining the antioxidant system and humoral immune system.

## INTRODUCTION

With the development of the poultry industry in China, chicken breeding and feed production are becoming more specialized and widespread. The poultry industry is not only concerned with the quality of life but also has a major impact on national economies. In the modern poultry industry, broilers are usually reared at a high stocking density to maximize kg of chicken produced per square meter of space to decrease the cost of production to achieve a satisfactory economic return. To our knowledge, a significant difference in the directive stocking density of broiler among various country. For example, stocking density with 45 to 54 kg/m^2^, 40 kg/m^2^, and 30 to 36 kg/m^2^ in Netherlands, Britain and Switzerland, respectively [1]. Moreover, the welfare guidelines of the National Chicken Council (NCC, 2005) recommend a density of 41.5 kg/m^2^ for broilers more than 2 kg in the USA [2]. In general, good environmental conditions should be provided for broilers to achieve their optimal growth properties [3,4]. Among those environmental conditions, stocking density is considered an important factor in broiler production because of its influences on broiler performance, welfare and health, as well as on well-being [5]. In addition, Dozier III [6] had reported that increasing the density beyond 30 kg/m^2^ elicited some negative effects on live performance of heavy broilers. Moreover, Simsek et al [7] indicated that crowding (up to 47.3 kg/m^2^) could lead to oxidative stress and a significant decrease in the bursa weight of broilers.

So, birds under high stocking density are more susceptible to infectious diseases because of the related physiological and immunological stress. It is well known that dietary modifications with certain antioxidant additives are among the most preferred and practical ways to ameliorate stress. Alpha-lipoic acid (ALA) is a sulfur-containing compound that functions as an essential coenzyme in carbohydrate metabolism in the mitochondria [8]. The ALA is both a fat- and water-soluble molecule and is able to scavenge free radicals that are not limited solely to one environment [9]. In *in vivo* systems, the ALA directly and indirectly regenerates other antioxidants, such as glutathione, ascorbate, and vitamin E [10]. Additionally, ALA is able to chelate a wide variety of metals that are associated with the increased production of free radicals [11]. In our previous study, ALA was added to the diet could relieve the negative influence of atmospheric ammonia stress [8]. Other research had reported that adding ALA to the broiler diet significantly increased glutathione content in the liver, decreased malondialdehyde (MDA) in serum [12]. Moreover, Li et al [13] found that the addition of 300 mg/kg LA to broiler diet could inhibit the adverse effects of aflatoxin B1.

Although many researches have been publishe d on the growth performance, antioxidant capability and physiological stress of dietary ALA under different environmental and/or dietary conditions [8,9,12], information on the relationship between stocking density and dietary ALA is sparse. Therefore, the present study was conducted to evaluate the impact of different stocking densities with or without ALA supplementation on the growth performance, physiological stress, immune response and changes of the antioxidant status in broilers.

## MATERIALS AND METHODS

### Animal care

This project was approved and conducted under the supervision of the Henan Academy of Agricultural Sciences Animal Care and Use Committee, which have adopted Animal Care and Use Guidelines governing all animal use in experimental procedures. All efforts were made to minimize suffering.

### Birds, diets and housing conditions

The study was conducted at the Institute of Animal Husbandry and Veterinary Science, Henan Academy of Agricultural Sciences (Zhengzhou), from May to June 2017. More than 4,000 one-day-old broilers (*Arbor Acers*) were obtained from a commercial hatchery (Wellhope Agri-Tech Joint Stock Co. Ltd., Kaifeng, China). To exclude the effect of sex, only male birds were used in this experiment. All birds were fed with a corn and soybean meal basal diet, which was formulated in mashed form, *ad libitum* for the first 21 days. On d 22, a total of 1,530 birds with comparable body weights (BW; 731.92± 5.26) were individually weighed and placed into 18 cages in groups of 75 (LD, low stocking density) birds, 90 (HD, high stocking density) birds and 90 birds with 300 mg/kg α-lipoic acid added to their basal diet (HD+ALA, high stocking density+ lipoic acid) [8,12]. Each treatment was represented by 6 replicates.

The chicks had been vaccinated for Marek’s disease, New castle and infectious bronchitis. The ALA was purchased from a pharmaceutical company (Xi’an Realin Biotechnology Co., Ltd., Xi’an, China). The basal diets (Table 1) were formulated to recommendations of the NRC [14]. The birds were reared in 6 three-tiered battery cages with wire floors. The length, width, and height of each cage was 2.46, 2.02, and 0.55 m, respectively. Thus, the cage floor area was approximately 5 m^2^ (2.46×2.02 m). The 2 levels of stocking density were calculated as 15 birds/m^2^ (37.5 kg/m^2^) for the low density group and 18 birds/m^2^ (45.0 kg/m^2^) for the high density group, based on a projected final BW of 2.5 kg [7].

The water and feeder spaces were similar in all cages and met or exceeded the recommendations for commercial practices. Each cage was provided with an automatic drinker (18 nipples for HD and 15 nipples for LD) and 2 feeding bins (2.40 m length each). The amount of feeding and drinking space available per bird was kept constant across the density treatments by blocking a proportion of the feeding area and drinking nipples within the cage. All birds were reared in an environmentally controlled house. The ventilation system in the house works by allowing air to enter the house through 2 humid walls located on both sides of the house. The outside air passes through the humid walls to cool and humidify the house. The ventilation was controlled to minimize contaminants such as dander, H_2_S, CO_2_ and ammonia in the house. The ambient temperature and relative humidity in the chambers were 35°C and 70% on the day of arrival and gradually decreased to a constant temperature and relative humidity of 24°C and 60%, respectively. The temperature was measured continuously in each pen at chick height. The light regime was 23 h of light and 1 h of dark.

### Sample preparation and parameter measurement

Individual BW was recorded at 1, 21, 35, and 42 d of age, and average daily feed intake (ADFI), average daily gain (ADG), and feed conversion ratio (FCR) were recorded weekly for each cage. Mortality was checked daily, and the weights of dead broilers were used to adjust FCR.

On d 35 and 42, four 12 h-fasted broilers from each cage were randomly selected from each group. A 4 mL blood sample was obtained from a wing vein and placed into 2 tubes (2 mL in each tube). Care was taken to ensure that the elapsed time between catching a bird and collecting the blood sample did not exceed 60 s. The first tube contained heparin to ensure that the blood sample was incoagulable before being transferred to the laboratory. One drop of blood from each heparinized tube was expelled to make a thin smear on a clean microscope slide. The dried blood smears were stained with Wright’s stain (Janssen Chimica, Beerse, Belgium), the leukocytes were microscopically differentially counted and the heterophil: lymphocyte (H:L) ratio was calculated.

The plasma from the remaining blood sample was obtained by centrifugation at 3,000×*g* for 10 min at 4°C and stored at −80°C for the immune function and antioxidant analyses. The activity of the total antioxidant capacity (T-AOC), superoxide dismutase (SOD), and glutathione peroxidase (GSH-Px), and the MDA content in the plasma were determined with clinical chemistry assay kits according to the manufacturer’s instructions (Nanjing Jiancheng Bioengineering Institute, Nanjing, China). The immune response status in serum was estimated by measuring the levels of immunoglobulin A (IgA), IgG, IgM, diamine oxidase (DAO), and interleukin-2. These indices were analyzed using the enzyme-linked immunosorbent assay method. All the details of the assays followed the manufacturer’s instructions for the commercial kits (Nanjing Jiancheng Bioengineering Institute, China).

On d 42, two 12 h-fasted birds were randomly selected from each cage, euthanized and manually exsanguinated. The spleen, thymus and bursa of Fabricius were immediately removed and weighed, and the absolute and relative weights (ratio of bursa or spleen weight to final BW) were used for statistical analysis. The breast and leg muscles were removed after deboning and weighed. Abdominal fat, composed of the fat tissues surrounding the proventriculus and gizzard lying against the inside abdominal wall and around the cloaca, was collected and weighed. After removing the heads and feet, the carcasses were eviscerated and weighed. The weight percentages of breast meat, leg meat, and abdominal fat were calculated as a percentage of eviscerated carcass weight.

### Statistical analysis

The data were analyzed by one-way analysis of variance (SPSS v20.0). Tukey’s multiple range test was employed to evaluate the differences, which were assumed to be statistically significant at p<0.05. The data in the tables are presented as the arithmetic mean and standard error of the mean of 6 replicates.

## RESULTS

### Growth performance and feed utilization of the broilers

The effects of stocking density and ALA on the growth performance, feed utilization and mortality of the broilers are shown in Table 2. No significant differences in BW were recorded up to 21 d of age (p>0.05). The mortality of each group was lower than 2% during the entire feeding period, and there was no significant difference among treatments (p = 0.625).

The results showed that the stocking density and ALA had no significant effect on BW, ADG, ADFI, and FCR on d 35 (p>0.05). However, the BW of the LD and HD+ALA groups were significantly higher than that of the HD group on d 42 (p<0.05). A mean reduction of approximately 5 g in ADG and 6 g in ADFI was observed in the HD group compared to the other groups on d 42 (p<0.05). The FCR at 42 d in the HD group was significantly higher (p = 0.020) compared to that of the LD and HD+ALA groups.

### Carcass performance and lymphoid organs of the broilers

In Table 3, the carcass weight and thigh yield were significantly lower in the HD group compared with those of the other groups on d 42 (p<0.05). The slaughter yield and carcass yield were approximately 90% and 72% of live BW, respectively. The breast yield as a percentage of carcass weight was approximately 20% for each of the treatments. The abdominal fat yield at 42 d in the HD+ALA group was significantly lower (p = 0.031) compared with that of the LD group. The spleen: BW and thymus:BW did not differ among those treatments (p>0.05); whereas bursa:BW significantly declined (p = 0.027) in the HD group on d 42.

### Stress indicators of the broilers

The effects of the stocking density and ALA on the stress indicators of the broilers are presented in Table 4. The stocking density and ALA were found to significantly affect the levels of serum SOD and GSH-Px (p<0.05), but no significant differences were found for T-AOC and MDA on d 35 (p>0.05). On d 42, the serum SOD and GSH-PX activity were dramatically decreased as stocking density increased (p<0.05); whereas the MDA concentration was increased compared with that of the LD group (p = 0.036). However, the addition of ALA suppressed the elevation of the serum MDA content (p = 0.036) and increased SOD (p = 0.026) and GSH-Px (p = 0.009) activity compared with those of the HD group (p<0.05) on d 42. The activity of T-AOC was not affected by treatment in this experiment on d 42 (p = 0.695).

### Immune response of the broilers

The effects of stocking density and ALA on the immune response of broilers are shown in Table 5. The stocking density and ALA did not have a significant effect on the immune response parameters except for IgA and DAO (p<0.05) on d 35. The serum concentrations of IgA and IgG were dramatically decreased as stocking density increased (p<0.05) on d 42. In addition, supplementation with 300 mg/kg of ALA in the diet inhibited the HD-mediated decrease in the serum titers of IgA and IgG (p<0.05). The level of DAO was significantly higher (p<0.001) in the HD group on d 42.

## DISCUSSION

### Growth performance of the broilers

Stocking density has critical implications for the broiler industry because higher returns can be obtained as the number of birds per unit of space increases. In this study, although the results indicated an improved growth performance and feed utilization in the LD group compared to those of the birds stocked at high density, the difference was not statistically significant on d 35. This may be due to the experimental period, which was carried out from d 22 to d 35. Similarly, Beloor et al [15] reported that stocking density did not influence the final body weight (FBW) and FI, which was attributed to the experimental period that lasted for only 5 weeks. Moreover, Shanawany [16] showed that stocking density did not significantly affect the FBW up to the age of 4 weeks, but it had a significant effect by the age of 5 and 6 weeks. However, at 42 d of age, the growth rate and FCR are inferior as the stocking density increased, but those differences were diminished when the broilers were fed ALA at the high stocking density. Some researches demonstrated that growth was suppressed at high stocking densities [17,18]. It is well known that housing conditions are paramount in intensive breeding [5] and increasing the stocking density could decrease the dissipation of body heat, enhance ammonia emissions and limit air circulation, which may be the causal factors leading to growth rate reductions.

In the present study, the ADFI decreased significantly in the HD group at 42 d. Shanawany [16] reported that stocking density could influence the feed consumption of broilers. Dozier III et al [6] found that the depression in BW gain due to stocking density was related to a reduction in feed consumption. A number of studies claimed that physical access of broilers to feeders and nipple waterers was limited by an increased stocking density, leading to reduced feed consumption [3,6,16]. However, the amount of feeding and drinking space available per bird was kept constant across density and met or exceeded the recommendations for commercial practices in this study. Hansen and Becker [19] demonstrated that even when constant feeding space per bird was maintained, the negative effects of density on final BW prevailed. Therefore, feeder and nipple accessibility cannot explain the changes in ADFI. Dawkins et al [5] reported that chickens that grew at the highest stocking densities jostled each other more and showed leg disease more than chickens at lower stocking densities, leading to difficulty moving for the broilers, and this result was probably responsible for the adverse effects of ADFI.

In the current study, dietary supplementation with ALA at the high stocking density had no significant difference on the performance traits compared with those of the LD group. Moreover, ALA had a positive effect on FCR during the finisher phase (22 to 42 d) of the experiment. These results indicate that ALA may prevent the growth-depressing effects of the high stocking densities and result in BWs equal to those found at the lower densities. This may be due to the ALA is a multifunction antioxidant, it can scavenge free radicals in poultry birds, and antioxidant supplementation in feed could improve the growth of broilers. Lu et al [12] had reported that ALA addition could relieve ammonia stress to restore broiler production performance to normal levels. Guo et al [20] indicated that ALA provision improved feed intake and weight gain on broilers. Similarly, Basmacıoğlu et al [21] also documented higher weight gain and feed conversion efficiency in broiler birds provided higher dietary α-tocopherol at the rate of 200 mg/kg concentration along with lipoic acid.

It is well known that under stressful conditions, the physiological metabolism of chickens will change, and their energy consumption will increase. Arshad et al [22] observed that the weight gain of broilers decreased significantly under stress, and antioxidant supplementation in feed improved the growth of the birds. Hooge [23] reported that diets supplemented with some kinds of prebiotics could ameliorate the performance of broilers under disease or crowing stress. Chen et al [12] found that adding ALA to a broiler diet could improve the antioxidant capability and reduce oxidative stress in broilers. However, El-Senousey et al [24] and Zhang et al [25] reported that dietary ALA can negatively impact the BW, ADG, and FI of broilers. The inconsistency in the effectiveness of ALA may be due to the effects of different factors. The Nutrition, housing condition, management, type of additive, dosage and bird characteristics can affect the response of broilers to ALA.

### Carcass yield and immune organs

In the present study, although carcass yield was not different between treatments, carcass weight was significantly lower in the higher compared to the lower stocking density. This result was consistent with the data of Tong et al [18] and Simitzis et al [4], who found that stocking density did not lead to apparent differences in carcass yield relative to BW. Moreover, the trial data showed that breast yield as a percentage of carcass weight ranged between 20.47% and 21.97%, and stocking density did not influence the breast yield significantly. This result was in accordance with some previous studies [6,26]. However, Feddes et al [3] stated that an increase in stocking density was expected to decrease breast muscle, as the more crowded chickens were not expected to grow to their full potential. Some studies determined that increasing the stocking density decreased breast fillet yield [27].

In this study, the thigh yield of chickens in the LD group was significantly higher compared with that of the HD group, which was consistent with Castellini et al [27], who reported that the percentage of thigh meat increased when birds had more forced motor activity at the lower stocking densities. Reiter and Bessei [28] reported that broilers given more exercise have less leg weakness. Furthermore, Sørensen et al [29] concluded that the lower stocking density substantially reduced the prevalence of leg weakness.

As shown in Table 3, dietary ALA decreased abdominal fat yield significantly compared with that of the low stocking density treatment in the present experiment. El-Senousey et al [24] observed that dietary ALA could reduce the abdominal fat weight of broilers by inhibiting body fat deposition. Similarly, Shen et al [30] showed that ALA decreased the percentage weight of abdominal fat in mice. This was probably due to ALA inhibiting adipocyte differentiation and indirectly promoting energy metabolism, for instance, accelerating fatty acid β-oxidation [31].

Under the conditions used in this study, neither density nor ALA appeared to affect the spleen:BW or thymus:BW, but the bursa:BW decreased with increasing stocking density of the supplementation of ALA improved the bursa:BW in the HD+ALA group. Dafwang et al [32] reported a significant decline in bursa weight with increasing stocking density, indicating that birds reared at high densities may be immunocompromised. Similarly, Simitzis et al [4] found a significant decrease in bursa weight at the higher stocking density and indicated that the stress levels increased at high stocking density. Different environmental factors can result in stress, including high stocking density, which leads to more conflicts and stress among broilers. Therefore, a decreased bursa weight is associated with increased levels of physiological stress. On the other hand, ALA has indirect favorable effects on antioxidant capability and oxidative stress [12]; as a consequence, the bursa:BW was not negatively affected by stocking density in the HD+ALA group. Nonetheless, a number of studies have found no evidence of an effect on lymphoid organs linked to high densities [17,18].

### Antioxidant capacity

It is thought that, under normal physiological states, a balance between antioxidant defenses and pro-oxidant production is immanent in living animals. Imbalance of this system will lead to elevation of reactive oxygen species (ROS) and induce oxidative stress. High level of ROS leads to oxidation and damage of lipids and proteins in the cell and cellular compartments. Therefore, living organisms are able to cope with oxidative stress by synthesize antioxidant enzymes and repair physiological systems. Antioxidant enzymes such as SOD, GSH, and GSH-Px play a vital role in antioxidant defense mechanisms [33]. Our data corresponded with the findings of Simitzis et al [4] and Simsek et al [7] shown that increasing stocking density resulted in the induction of oxidative stress status in broilers, as was evident by increased MDA and reduced GSH-Px and SOD activity in the blood serum, whereas no differences were observed in serum T-AOC activity between treatments.

The MDA is the main final product of lipid peroxidation and is always evaluated as a biomarker of oxidative damage [34]. In the current study, the serum MDA level in the HD group was significantly higher than that in the LD group. This result may be due to crowding increasing conflicts among chickens and causing stress, thereby resulting in higher lipid peroxidation. Similarly, Simsek et al [7] indicated that crowding enhanced oxidative destruction and caused MDA generation. Taken together, it could be suggested that, under a high stocking density, the elevated lipid peroxidation from stress could result from the decreased activity of antioxidant enzymes. Therefore, a reduction in oxidative stress becomes more important for maintaining normal animal production.

Several studies have reported that the addition of moderate dietary antioxidants could provide the poultry industry with a simple and practical method for improving oxidative stability [35]. In recent years, an increasing number of studies have noted the antioxidant benefits of ALA [36]. In general, lipoic acid prevents and treats some diseases through its antioxidant and anti-inflammatory actions, as it is capable of scavenging hydroxy radicals, hypochlorous acid and singlet oxygen [11]. In the present study, the results showed that ALA supplementation significantly depressed the MDA content and increased GSH-PX and SOD activity in the serum of the high stocking density treatment. Similar results have been found by Lu et al [8], who claimed that ALA supplementation increased SOD and GSH-Px activity and decreased MDA content in serum to different extents under ammonia stress; Ma et al [37] reported that ALA in the diet could increase the hepatic GSH-Px gene expression under aflatoxin B1-induced liver oxidative damage. These results suggested that ALA could ameliorate the antioxidant status of broilers and mitigate oxidative stress caused by a higher stocking density. This may be due to exogenously supplied ALA is readily taken up by a variety of cells and tissues in which it is rapidly reduced by nicotinamide adenine dinucleotide or nicotinamide adenine dinucleotide phosphate—dependent enzymes to dihydrolipoic acid [11]. Additionally, ALA is a potent direct reactive species quencher in its oxidized form. Moreover, ALA can effectively enhance the antioxidants enzymes concentrations by providing the reducing substrate and regenerating them via the reduction of their radicals [38].

### Immune response

The immune system mainly involves humoral immunity and cell-mediated immunity. It is well known that the immune system is very sensitive to animal health. In the present study, although mortality was not affected by increasing stocking density or supplementing ALA, the significant decline in serum globulin titers (IgG and IgA) with increasing stocking density suggest that broilers reared at high densities may be immunocompromised. Similarly, Vo and Fanguy [39] found that high densities suppressed humoral immunity and increased mortality. Moreover, as Table 5 shows, the IgA and IgG serum concentrations increased greatly with the addition of ALA at the high stocking density; these results suggest that stocking density may affect the humoral immune response and that ALA actively inhibits the HD-mediated impairment of Ig production.

As well known, dysfunction in this barrier can occur under several types of stress, such as psychological, physiological and pathological stress [40]. In the present study, stocking density could induce a change in physiological stress and an increase in the peroxide level in broilers [4,7]. Additionally, Kim et al [41] found that stress could lead to impaired mucosal barrier function and increased intestinal permeability during early weaning of pigs. A notable result from this study was that DAO significantly increased in the HD group and then returned to a normal level with the dietary addition of 300 mg/kg ALA, which indicates that ALA could improve the intestinal health of physiologically stressed broilers. Similarly, Lei et al [42] reported that a decrease in serum DAO activity reflected an amelioration of intestinal barrier function in chickens.

In the present study, a moderate (not significantly) effect of stocking density on H:L ratio was found. Similar results have been reports in H:L ratio between different stocking densities [43], whereas Simitzis et al [4] found a trend for increasing H:L ratio with increasing densities. It is possible that the observed difference between those researches in H:L ratio could be the result of a combination of genotype and environment factors [43], since H:L ratio can change quite rapidly due to various stressors.

## CONCLUSION

In conclusion, the stocking density showed no significant effect on the performance parameters and feed utilization on d 35. However, the increase in stocking density decreased the growth performance, FCR and carcass traits; induced oxidative stress and immunodeficiency; and caused injury the intestinal function of broilers at 42 d. In addition, supplementation with 300 mg/kg ALA in the diet inhibited body fat deposition and eliminated those negative effects in the high stocking density.

## Figures and Tables

**Table 1:** Composition and nutrient levels of the basal diet (air-dry basis)

Items	Starter (0 to 21 d)	Finisher (22 to 42 d)
Ingredients (%)		
Corn	57.30	61.60
Soybean meal	37.80	33.50
Soybean oil	1.90	2.50
Salt	0.30	0.30
Limestone	1.65	1.58
Choline chloride	0.10	0.10
CaHPO_4_	0.44	0.15
Vitamin premix1)	0.02	0.02
Mineral premix2)	0.20	0.20
L-lysine	0.08	0.02
DL-methionine	0.19	0.10
Phytase	0.02	0.02
Total	100.00	100.00
Nutrient composition (%)		
Crude protein	21.50	20.05
Calcium	1.02	0.91
Available phosphorus	0.46	0.41
Lysine	1.15	1.00
Methionine	0.52	0.42
Methionine+cysteine	0.85	0.77
Threonine	0.88	0.81
Tryptophan	0.29	0.27
Apparent metabolizable energy (MJ/kg)	12.57	12.99

1) Vitamin premix (per kilogram of diet): vitamin A, 12,000 IU; vitamin D_3_, 2,000 IU; vitamin E, 20.75 mg; vitamin K_3_, 2.65 mg; vitamin B_1_, 2 mg; vitamin B_2_, 5 mg; vitamin B_6_, 2 mg; vitamin B_12_, 0.025 mg; biotin, 0.0325 mg; folic acid, 1.25 mg; pantothenic acid, 12 mg; niacin, 50 mg.

2) Mineral premix (per kilogram of diet): Mn, 100 mg; I, 0.35 mg; Se, 0.15 mg; Zn, 75 mg; Cu, 8 mg; Fe, 80 mg; Co, 0.2 mg.

**Table 2:** Effect of stocking density and alpha-lipoic acid on the growth performance and mortality of broilers at different ages

Items	Age (d)	Treatments1)			SEM	p-value

		LD	HD	HD+ALA		
BW (g)	21	732	726	736	3.26	0.473
	35	1,911	1,858	1,930	17.39	0.232
	42	2,600^a^	2,471^b^	2,593^a^	21.13	0.004
ADG (g/d)	22 to 35	84	80	85	1.19	0.319
	22 to 42	88^a^	83^b^	88^a^	0.92	0.002
ADFI (g/d)	22 to 35	135	130	133	1.06	0.147
	22 to 42	155^a^	148^b^	153^ab^	1.16	0.025
FCR (g/g)	22 to 35	1.61	1.62	1.57	0.02	0.487
	22 to 42	1.75^b^	1.79^a^	1.73^b^	0.01	0.020
Mortality (%)	22 to 42	1.63	1.93	1.38	0.22	0.625

Values are means, n = 6 per treatment.

SEM, standard error of the mean; BW, body weight; ADG, average daily gain; ADFI, average daily feed intake; FCR, feed conversion ratio.

1) LD, low stocking density (15 birds/m^2^); HD, high stocking density (18 birds/m^2^); HD+ALA, high stocking density+α-lipoic acid (18 birds/m^2^+300 mg/kg).

a,b Different letters in the same row indicate significant differences (p<0.05).

**Table 3:** Effect of stocking density and alpha-lipoic acid on the carcass performance and lymphoid organs of broilers at 42 d

Items	Treatments1)			SEM	p-value

	LD	HD	HD+ALA		
Carcass performance					
Slaughter yield2) (%)	91.44	90.23	90.16	0.31	0.179
Carcass weight (g)	1,884^a^	1,791^b^	1,869^a^	16.62	0.027
Carcass yield2) (%)	72.47	72.47	72.11	0.39	0.923
Breast yield3) (%)	20.47	20.09	21.97	0.40	0.117
Thigh yield3) (%)	19.37^a^	18.41^b^	18.79^ab^	0.16	0.028
Abdominal fat yield3) (%)	2.27^a^	2.00^ab^	1.84^b^	0.07	0.031
Immune organ index2) (g/kg BW)					
Spleen:BW	1.29	1.24	1.25	0.08	0.695
Thymus:BW	4.90	5.07	4.94	0.10	0.793
Bursa:BW	1.83^a^	1.75^b^	1.85^a^	0.02	0.027

Values are means, n = 6 per treatment.

SEM, standard error of the mean; BW, body weight.

1) LD, low stocking density (15 birds/m^2^); HD, high stocking density (18 birds/m^2^); HD+ALA, high stocking density+α-lipoic acid (18 birds/m^2^+300 mg/kg).

2) Calculated as a percentage of live BW.

3) Calculated as a percentage of carcass weight.

a,b Different letters in the same row indicate significant differences (p<0.05).

**Table 4:** Effect of stocking density and alpha-lipoic acid on antioxidative ability in serum of broilers at different ages

Items	Treatments1)			SEM	p-value

	LD	HD	HD+LA		
35 d					
T-AOC (U/mL)	9.03	9.11	9.65	0.14	0.158
SOD (U/mL)	244.41^a^	225.00^b^	236.68^a^	2.98	0.009
GSH-Px (U/mL)	410.46^a^	382.19^b^	417.35^a^	5.44	0.004
MDA (nmol/mL)	1.92	2.18	2.06	0.09	0.537
42 d					
T-AOC (U/mL)	12.07	11.70	12.36	0.29	0.695
SOD (U/mL)	273.01^a^	252.81^b^	279.52^a^	4.60	0.026
GSH-Px (U/mL)	432.51^a^	412.24^b^	447.79^a^	5.46	0.009
MDA (nmol/mL)	2.00^b^	2.29a	1.95^b^	0.06	0.036

Values are mean, n = 6 per treatment.

SEM, standard error of the mean; T-AOC, total antioxidant capacity; SOD, superoxide dismutase; GSH-Px, glutathione peroxidase; MDA, malondialdehyde.

1) LD, low stocking density (15 birds/m^2^); HD, high stocking density (18 birds/m^2^); HD+ALA, high stocking density+α-lipoic acid (18 birds/m^2^+300 mg/kg).

a,b Means within the same row with different superscripts differ significantly (p<0.05).

**Table 5:** Effect of stocking density and alpha-lipoic acid on the immune response in the serum of broilers at different ages

Items	Treatments1)			SEM	p-value

	LD	HD	HD+ALA		
35 d					
IgG (mg/mL)	3.78	3.56	4.07	0.17	0.512
IgA (mg/mL)	0.73^ab^	0.67^b^	0.79^a^	0.02	0.025
IgM (mg/mL)	1.33	1.20	1.27	0.06	0.024
DAO (μg/L)	0.95^b^	1.16^a^	0.83^b^	0.05	0.004
IL-2 (ng/L)	1.72	1.81	1.73	0.03	0.421
H:L ratio	0.47	0.46	0.44	0.02	0.716
42 d					
IgG (mg/mL)	4.68^a^	4.12^b^	4.84^a^	0.13	0.031
IgA (mg/mL)	0.80^ab^	0.68^b^	0.92^a^	0.04	0.016
IgM (mg/mL)	1.39	1.32	1.43	0.04	0.607
DAO (μg/L)	0.96^b^	1.20^a^	0.85^c^	0.05	0.000
IL-2 (ng/L)	1.99	1.92	1.87	0.03	0.306
H:L ratio	0.45	0.43	0.40	0.01	0.205

Values are means, n = 6 per treatment.

SEM, standard error of the mean; IgG, immunoglobulin G; IgA, immunoglobulin A; IgM, immunoglobulin M; DAO, diamine oxidase; IL-2, interleukin-2; H:L ratio, heterophil:lymphocyte ratio.

1) LD, low stocking density (15 birds/m^2^); HD, high stocking density (18 birds/m^2^); HD+ALA, high stocking density+α-lipoic acid (18 birds/m^2^+300 mg/kg).

a–c Different letters in the same row indicate significant differences (p<0.05).
